# Chemo brain or tumor brain - that is the question: the presence of extracranial tumors profoundly affects molecular processes in the prefrontal cortex of TumorGraft mice

**DOI:** 10.18632/aging.101243

**Published:** 2017-07-29

**Authors:** Anna Kovalchuk, Yaroslav Ilnytskyy, Rocio Rodriguez-Juarez, Svitlana Shpyleva, Stepan Melnyk, Igor Pogribny, Amanda Katz, David Sidransky, Olga Kovalchuk, Bryan Kolb

**Affiliations:** ^1^ Department of Neuroscience, University of Lethbridge, Lethbridge, AB T1K 6T5, Canada; ^2^ Department of Biological Sciences, University of Lethbridge, Lethbridge, AB T1K 6T5, Canada; ^3^ Division of Biochemical Toxicology, National Center for Toxicological Research, FDA, Jefferson, AR 72079, USA; ^4^ Department of Pediatrics, University of Arkansas for Medical Sciences, Little Rock, AR 72205, USA; ^5^ Department of Oncology, Champions Oncology, Baltimore, MD 21205, USA; ^6^ Leaders in Medicine Program, Cumming School of Medicine, University of Calgary, Calgary, T2N 1N4, Canada

**Keywords:** chemo brain, tumor brain, gene expression, DNA methylation, aging

## Abstract

Cancer chemotherapy causes numerous persistent central nervous system complications. This condition is known as chemo brain. Cognitive impairments occur even before treatment, and hence are referred to as *cancer associated cognitive change*s, or tumor brain. There is much yet to be learned about the mechanisms of both chemo brain and tumor brain. The frequency and timing of chemo brain and tumor brain occurrence and persistence strongly suggest they may be epigenetic in nature and associated with altered gene expression. Here we used TumorGraft^TM^ models wherein part of a patient's tumor is removed and grafted into immune-deficient mice and conducted global gene expression and DNA methylation analysis. We show that malignant non-central nervous system tumor growth causes profound molecular alterations in the brain. Mice harbouring triple negative or progesterone positive breast cancer TumorGrafts exhibited altered gene expression, decreased levels of DNA methylation, increased levels of DNA hydroxymethylation, and oxidative stress in the prefrontal cortex. Interestingly, chemotherapy did not have any additional synergistic effects on the analyzed processes. The molecular changes observed in this study are known signs of neurodegeneration and brain aging. This study provides an important roadmap for future large-scale analysis of the molecular and cellular mechanisms of tumor brain.

## INTRODUCTION

Breast cancer is one of the most commonly diagnosed cancers in the world, and it is the most common cause of cancer-related deaths in women. According to the Canadian Cancer Society in 2015, breast cancer accounted for 26% of all new cancer cases in Canadian women. Most breast cancer patients undergo chemo-therapy treatments, and the development of new chemotherapy regimens resulted in significant improvement of patient outcomes and increased survival. Despite the undisputed benefits, chemotherapy causes an array of side effects, such as myelosuppression, nausea, vomiting, infections, and bleeding. Additionally, many patients experience profound psychosocial effects that decrease their quality of life, including fatigue, cognitive dysfunction, and other signs of central nervous system (CNS) toxicity post-chemotherapy [1]. Thus, chemotherapy-induced cognitive changes have become an increasing concern among cancer survivors. Survivors have coined the terms *chemo fog* or *chemo brain* to explain their symptoms [2]. While the initial reports of chemo brain go back to the 1970s and the mid-1980s, the problem started to gain attention only in the ‘90s. Since then, numerous longitudinal and cross-sectional studies have reported the existence of chemo brain and its severity. Among these, the vast majority of work was conducted in breast cancer cohorts. It has been found that chemotherapy-induced CNS side effects, or chemo brain, affect almost half of all breast cancer survivors and impacts the cognitive domains of attention, memory, psychomotor speed, and executive function. Current research shows that chemotherapy agents are more toxic to healthy brain cells than to cancer cells, and debilitating chemo brain manifestations affect patients for as long as five to ten years after treatment completion [3–6].

To prevent and mitigate chemo brain side effects, it is important to understand the underlying neural mechanisms that are altered by chemotherapy agents. At present, mechanistic data on chemo brain are scarce. While some molecular mechanisms underlying chemo brain have been assessed in clinical studies, analyses are difficult to conduct because of large inter-patient variability, different treatment protocols, disease statuses, and co-morbidities [2]. Thus, recent chemo brain research has employed cell lines as well as rodent models in which healthy animals are treated with chemotherapy drugs. In these animal models, chemo-therapy caused oxidative stress, inhibited neuronal proliferation and differentiation, induced apoptosis, and altered levels of histone modification and chromatin remodeling, leading to the aberrant levels of neuro-trophic and neurogenic proteins [7, 8]. These molecular changes were associated with altered neuro-genesis and deficits in learning and memory processes [7, 9, 10].

Interestingly, recent data based on thorough pre-treatment assessments have revealed that 20–30% of breast cancer patients exhibit reduced cognitive performance prior to chemotherapy treatment, and this cognitive impairment was not related to surgery, fatigue, depression, or anxiety associated with their breast cancer diagnoses and treatments. Instead, these symptoms were correlated with the presence of the malignant tumor. While earlier studies suggested that cognitive impairment was due to chemotherapy, recent evidence of pre-treatment cognitive deficits led to a new term, “*cancer and cancer treatment-associated cognitive change”* [1]. This phenomenon may thus be referred to as *tumor brain*.

While the molecular and cellular mechanisms of chemo brain are poorly investigated, and the mechanisms of tumor brain have not even been looked at, the frequency, timing, and persistent nature of these phenomena strongly suggest they may be epigenetic. Epigenetic changes are meiotically heritable and mitotically stable alterations that regulate gene expression and genome stability; they include DNA methylation and hydroxymethylation, histone modification, and non-coding RNA regulation [11]. Epigenetic changes underlie aberrant global gene expression patterns, and they are critical to neurogenesis and CNS development and functioning [12]. Furthermore, chemotherapy drugs may alter epigenetic homeostasis and gene expression [13]. Epigenetic changes that underlie aberrant gene expression patterns have been well-documented in breast cancer literature. Recently, we analysed the molecular mechanisms of chemo brain [14] by investigating the gene expression profiles in the prefrontal cortex (PFC) and hippocampus (HPC) of mice 3 weeks after treatment with the cytotoxic chemotherapy agents mitomycin C (MMC) and cyclophosphamide (CPP) [14]. We showed that chemotherapy altered gene expression profiles in the PFC and HPC tissues; the changes were most prominent in the PFC tissues of females 3 weeks after MMC treatment. MMC exposure led to oxidative stress, decreased global DNA methylation and increased DNA hydroxymethylation in the PFC tissues of females. This opened new avenues for the analysis of epigenetic mechanisms of chemo brain [14].

Nonetheless, all data on chemo brain, including ours, stem from models in which healthy animals are treated with chemotherapy drugs. These models lack one important biological component—the presence of a tumor. To gain a complete understanding of the molecular mechanisms and pathways affected in tumor brain and chemo brain, we use TumorGraft^TM^ models whereby tumor tissue is engrafted into immune-deficient [15, 16]. TumorGraft technology is used as a cutting-edge personalized approach to cancer therapy. It preserves the characteristics of the live tumor, creating a replica that is identical to the tumor in the patient's body [17]. TumorGrafts maintain the characteristics of the tumor, including all tumor cells and supportive stroma. They are excellent representations tumor tissue in vivo, and therefore are effectively used for research and precision medicine [18].

Our study is the first to show that non-CNS malignant tumor growth causes profound molecular alterations in the prefrontal cortex, a key regulatory region that is involved in executive functions, such as working memory, decision-making, planning, judgment, social behavior, as well as abstract thinking.

## RESULTS

### Breast cancer growth affects global gene expression in prefrontal cortex tissue of tumor-bearing mice

Analysis of differential gene expression: Global transcriptome reflects all expressed mutational and non-mutational changes, and hence it is one of the best representations of molecular processes in cells and tissues. Global transcriptomic profiling constitutes an excellent tool to dissect underlying mechanisms of various diseases and conditions, as well as treatment responses. To gain a complete understanding of the effects of non-CNS tumors and chemotherapy on the brain, we used the Illumina next generation sequencing platform to perform an in-depth transcriptome analysis of the PFC tissue of TumorGraft mice with either triple negative breast cancer (TNBC) or progesterone receptor positive breast cancer (PR+).

**Figure 1:**
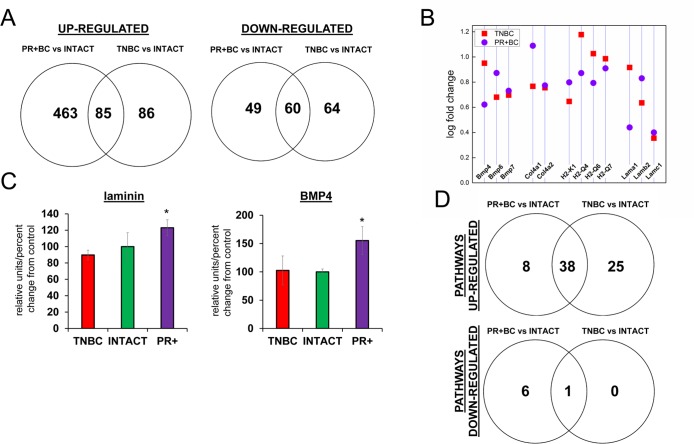
Next generation sequencing‐based analysis of gene expression in the PFC tissues of intact and TNBC and PR+Bcbearing TumorGraft mice (**A**) Venn diagram showing genes that were significantly different between TNBC and PR+BC mice, as compared to intact controls; (**B**) Fold changes in the levels of expression of selected genes; (**C**) Western immunoblotting analysis of laminin and BMP4 proteins in the PFC tissues of TNBC and PR+BC mice; data are shown as relative units/percent change of control. Due to size difference the same membrane was used for both proteins. * p<0.05, Student's t‐test; (**D**) Summary of molecular pathways that were altered in the PFCs of TNBC and PR+BC mice as compared to intact controls. The Pathview/KEGG analysis was used to determine differentially affected pathways.

The differential gene expression analysis revealed notable changes in PFC tissues of TNBC and PR+ animals (Fig. 1A). Hierarchical clustering of the gene expression data showed that each experimental group could be distinguished by its gene expression profile. Furthermore, the principal component analysis based on the entire transcriptome dataset showed good clustering for each group and clear differences between gene expression profiles in the PFC tissues of intact, TNBC and PR+BC TumorGraft mice (Fig. 1S).

In the PFC tissues of TNBC animals, 171 genes were upregulated, and 124, downregulated, as compared to intact mice. In the PFC tissues of PR+BC PDX mice, 548 genes were upregulated, and 109 were down-regulated as compared to intact animals. Amongst those, 85 were upregulated, and 60 were downregulated in the PFC tissues of both TNBC and PR+BC PDX mice as compared to intact animals (the adjusted p-value was <0.05; the fold change was 1.5) (Fig. 1A) Commonly upregulated genes included bone morphogenetic protein (*BMP*) and collagen genes, laminins, and histocompatibility loci (Fig. 1B). Laminin1-2 and BMP4 upregulation was also confirmed on the protein level in the PFC tissues of PR+BC mice, but not in TNBC mice (Fig. 1C).

To gain further insight into the functional significance of the observed transcriptome changes, we conducted an in-depth pathway analysis, during which we performed a functional annotation of differentially expressed genes using the Pathview/KEGG and DAVID bioinformatics platforms. One-directional pathway analysis revealed multiple differentially affected pathways. Amongst those, 46 pathways were upregulated in the PFC tissues of the PR+BC animals and 63 pathways – in the TNBC animals. Of those, 38 pathways were common to both tumor groups and included pathways involved in graft−versus−host disease, natural killer cell-mediated cytotoxicity, oxidative phosphorylation, as well as other pathways implicated in the inflammation and immune responses. In addition, one pathway was downregulated in the PFC tissues of TNBC animals, and 7 pathways in the PR+BC harboring animals. The neuroactive ligand pathway was common for both aforementioned groups (Fig. 1 D).

### Oxidative damage in the PFC tissues of tumor-bearing mice

**Figure 2:**
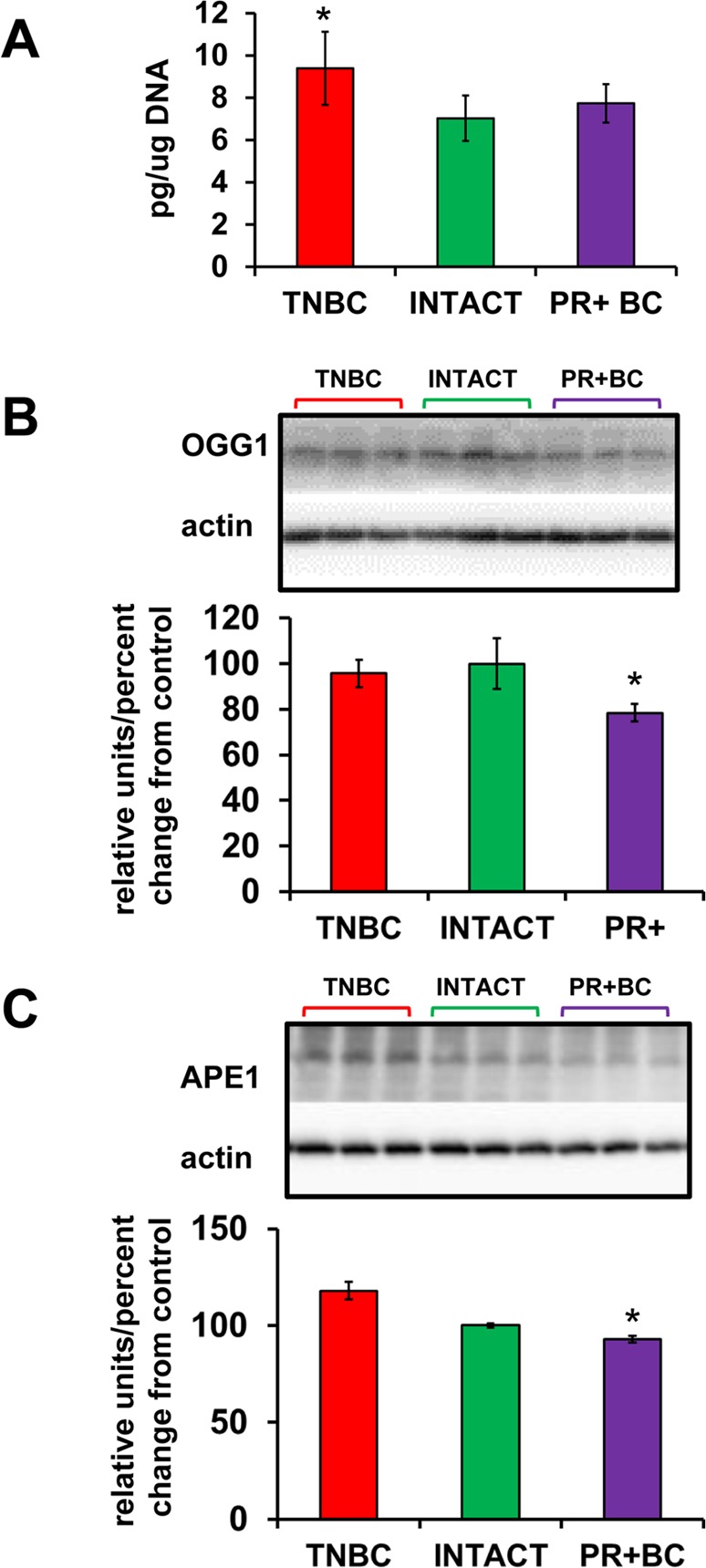
Oxidative DNA damage in PFC tissues of intact and TNBC and PR+BC-bearing TumorGraft mice (**A**) Levels of 8-oxo-7-hydrodeoxyguanosine (8-oxodG) in genomic DNA isolated from PFC tissues (mean ± SD, n=3 for INTACT and PR+BC mice; n=4 for TNBC mice); (**B**) Western immunoblotting analysis of the base excision repair protein OGG1; data are shown as relative units/percent change from control. Due to protein size differences and scarcity of tissue, membranes were re-used several times. * - significantly different from control mice, p<0.05, Student's t-test.

We noted alterations in the oxidative phosphorylation pathways in the PFC tissues of PDX mice. Oxidative stress is a hallmark of cancer. Previous studies, including our own, have shown increased oxidative stress in chemo brain [11, 19]. With this in mind, we analyzed the levels of 8-oxo-2′-deoxyguanosine (8-oxodG) in genomic DNA from the PFC tissues of TNBC- and PR+BC PDX–harboring animals. The 8-oxodG is one of the predominant and best-studied markers of oxidative DNA damage. It is formed by the action of reactive oxygen species [20]. The growth of a non-CNS TNBC tumor caused a strong and statistically significant (p=0.0472) increase in the 8-oxodG levels in the PFC tissues of TumorGraft animals (Fig. 2A). However, PR+BC tumor growth did not cause any significant increase in the 8-oxodG levels in the PFC of TumorGraft mice. We also determined levels of 8-oxoguanine glycosylase (OGG1) and apurinic/apyrimidinic endonuclease 1 (APE1), the base excision repair proteins that partake in the repair of oxidative DNA damage and constitute well-accepted markers of oxi-dative DNA damage [21, 22]. Western immuno-blotting revealed a statistically significant reduction in the levels of OGG1 (p=0.0039) and APE1 (p=0.033) in the PFC tissues of PR+BC mice (Fig. 2B).

### Global DNA methylation and level of DNA methyltransferases and methyl-CpG-binding protein MeCP2 in the PFC tissues of tumor-bearing mice

**Figure 3:**
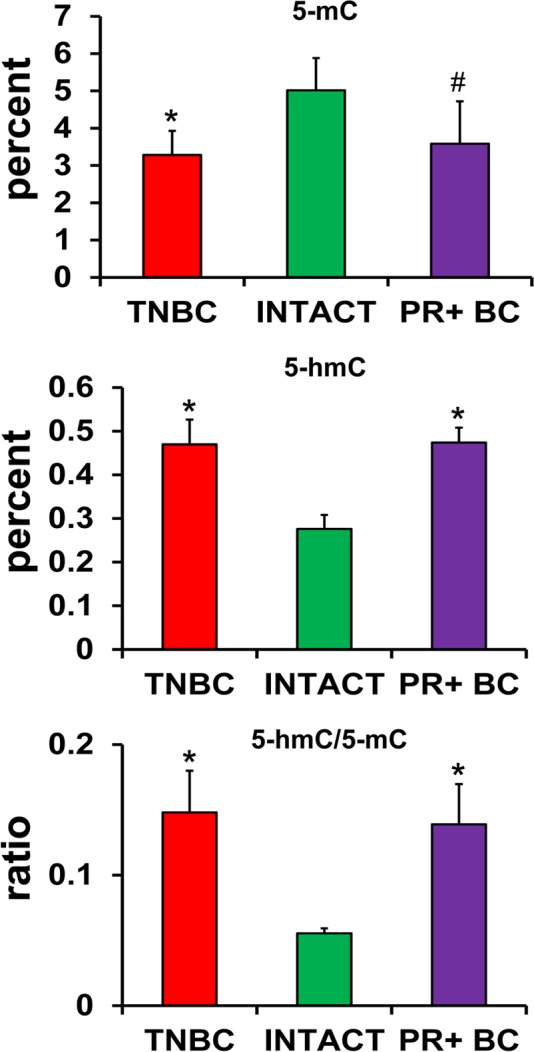
Levels of 5-mC and 5-hmC and ratio of 5-hmC/5-mC in the genomic DNA of PFC tissues of intact and TNBC and PR+BC-bearing TumorGraft mice * p<0.05, # p<0.10, Student's t-test.

Several studies, including our own, have suggested that aberrant DNA methylation may occur because of oxidative DNA damage [21]. Aberrant DNA methylation is also associated with altered gene expres-sion patterns [23, 24]. We analyzed and compared the status of global DNA methylation in the PFC tissues of TNBC and PR+BC PDX mice. Both 5-hydroxymethyl-cytosine (5-hmC) and 5-methyl-cytosine (5-mC) have recently emerged as important epigenetic markers. In order to get a complete account of global DNA methylation, we determined the levels of 5-mC and 5-hmC, as well as the ratio between them, in the genomic PFC DNA of intact and tumor-bearing animals. We found a statistically significant decrease in 5-mC levels in the global DNA of PFC tissues of TNBC-bearing animals (p=0.014), as well as a trend toward a decrease (90% confidence level, p=0.078) in the PFC tissues of PR+BC PDX animals as compared to intact controls. While the levels of 5-mC were reduced, the levels of 5-hmC were significantly increased in the PFC tissues of TNBC- and PR+BC-bearing mice (p=0.0017 and p=0.0009, respectively) as compared to intact controls. The ratio between 5-hmC and 5-mC was also changed in the PDX animals and was significantly increased in both TNBC and PR+BC PDX animals as compared to controls (Fig. 3).

**Figure 4:**
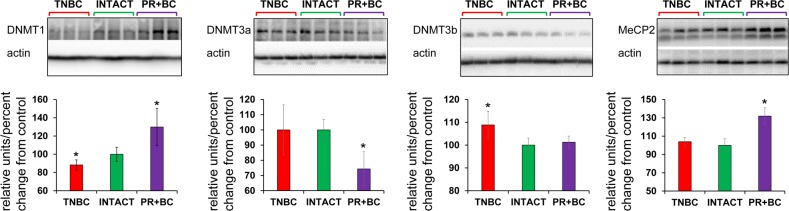
Levels of DNA methyltransferases DNMT1 and DNMT3a, and methyl-CpG binding protein MeCP2, in in the PFC tissues of intact and TNBC and PR+BC-bearing TumorGraft mice Data are shown as relative units/percent change of control. Due to protein size differences and scarcity of tissue, membranes were re-used several times. Significantly different from control mice - * p<0.05, # p<0.10, Student's t-test.

Having observed altered DNA methylation, we then determined the levels of DNA methyltransferases (DNMT1, DNMT3A and DNMT3B), as their altered levels may be associated with changes observed in 5-mC and 5-hmC levels. We noted that levels of DNMT1 were significantly (p=0.028) reduced in the PFC tissue of the TNBC PDX mice, but increased – in the PFC of the PR+BC mice (p=0.028). The levels of DNMT3A were decreased in the PFC tissues of PR+BC PDX animals (p=0.021), and unchanged in the PFC of TNBC mice. The levels of DNMT3B were unchanged in the PFC of PR+BC tissues, but upregulated in TNBC-bearing mice as compared to intact controls (p=0.021). At the same time, the levels of methyl-CpG-binding protein MeCP2 were significantly elevated (p=0.005) in the PFC tissues of PR+BC animals, and unaffected in those of TNBC mice as compared to intact control animals (Fig. 4).

### Effects of chemotherapy treatments on the molecular processes in the PFC tissues of tumor-bearing animals

**Figure 5:**
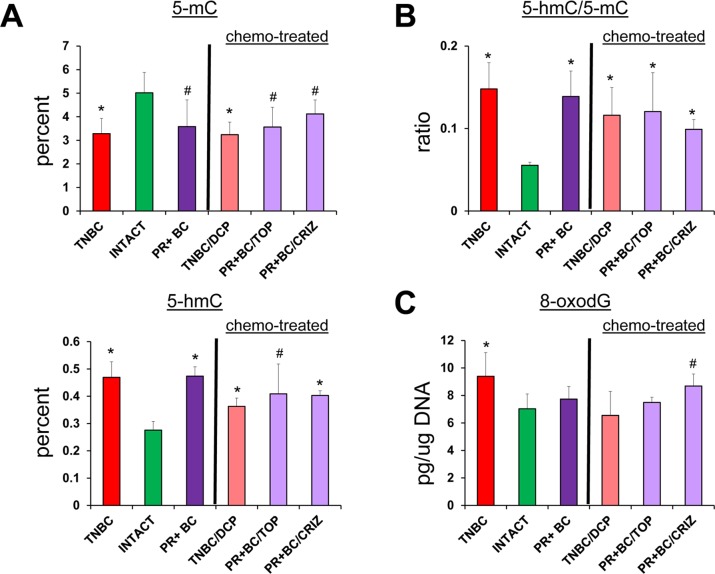
Tumor vs. chemo brain – levels of 8-oxodG, 5-mC and 5-hmC, and ratio of 5-hmC/5-mC in genomic DNA isolated from the PFC tissues of TNBC and PR+BC-bearing chemotherapy treated and untreated TumorGraft mice Significantly different from control mice -* p<0.05, # p<0.10, Student's t-test.

In order to establish whether or not chemotherapy treatments will further exacerbate tumor-induced molecular epigenetic changes in the PFC tissues of tumor-bearing animals, we analyzed the levels of genomic DNA methylation and oxidative stress marker 8-oxod-G in the PFC tissues of tumor-bearing and chemotherapy treated animals. Interestingly, chemotherapy treatments did not act in synergy with the 5-mC, 5-hmC and 8-oxo-dG changes induced tumor presence alone (Fig. 5).

**Figure 6:**
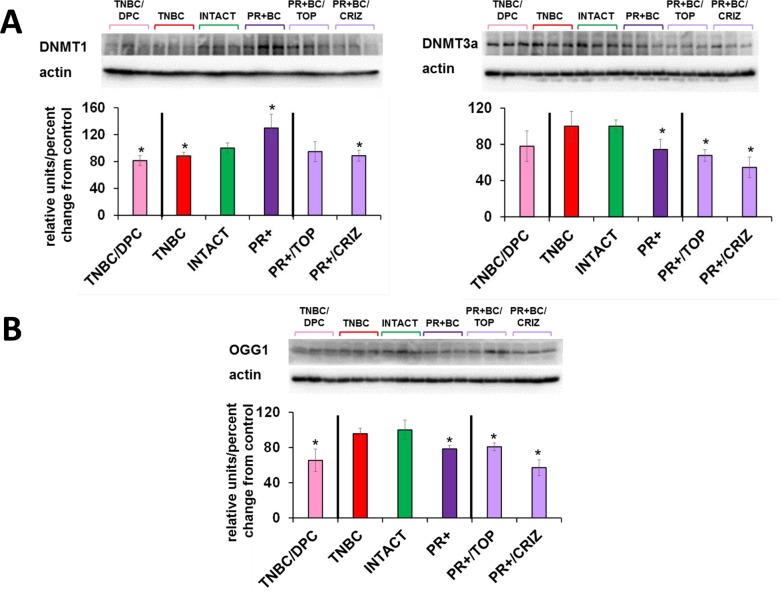
Levels of DNA methyltransferases DNMT1 and DNMT3a, methyl-CpG binding protein MeCP2, and oxidative damage repair protein OGG1, in in the PFC tissues of intact and TNBC and PR+BC-bearing chemotherapy treated and untreated TumorGraft mice (**A**) DNA methyltransferases and MeCP2 protein; (**B**) OGG1 protein. Data are shown as relative units/percent change of control. Data from chemotherapy-treated animals are shown along with intact controls and tumor-bearing untreated animals (also see Fig. 2B and 4). Due to protein size differences and scarcity of tissue, membranes were re-used several times. Significantly different from control mice -* p<0.05, # p<0.10, Student's t-test.

Chemotherapy treatments also affected the levels of DNMT1 and DNMT3A in the PFC tissues of tumor-bearing animals. There, doxorubicin-pactitaxel-cyclo-phosphamide (DPC) treatment of TNBC-bearing mice led to a statistically significant (p=0.024, as compared to intact controls) decrease in the levels of DNMT1 in the PFC tissues, and to an insignificant trend towards a decrease in DNMT3A. While the presence of PR+BC caused an increase in the DNMT1 levels in the PFC tissues of PDX animals as compared to controls, crizotinib chemotherapy led to a significant, albeit small, decrease in the DNMT1 levels. Furthermore, topotecan and crizotinib chemotherapies led to significant (p=0.021 and p=0.004, respectively, as compared to intact animals) decreases in the levels of DNMT3A. Moreover, crizotinib treatment of PR+BC mice furthered the decrease of DNMT3a levels in the PFC tissues as com-pared to untreated PR+BC mice (p=0.0058) (Fig. 6A).

Interestingly, chemotherapy treatments (DPC for TNBC and crizotinib and topotecan for PR+BC) strongly affect-ed OGG1 levels, causing statistically significant decrea-ses in the levels of this DNA repair protein (Fig. 6B).

## DISCUSSION

CNS toxicity is one of the major quality-of-life issues that cancer survivors face. Nevertheless, there is a lot to learn about the mechanisms of chemo brain [1, 25]. Along with chemo brain, studies have emerged reporting notable cognitive changes and memory deficits prior to chemotherapy or other cancer treat-ments, the ‘tumor brain’ phenomenon [1, 4, 25].

This is the first study showing that non-CNS malignant tumor growth causes profound molecular alterations in the brain. Our key findings were that:

- the growth of malignant non-CNS tumors profoundly affected the brain and exerted a negative influence on the PFC;
- PDX mice carrying TNBC and PR+BCX PDXs exhibited altered gene expression in the PFC;
- the growth of TNBC and PR+BC tumors caused oxidative stress and aberrant DNA methylation in the PFC tissues of PDX mice; and
- chemotherapy treatments did not have any additional synergistic effects on the analyzed processes.

We observed profound changes in the global gene expression in the prefrontal cortex of PDX-carrying mice. We found the upregulation of laminin, bone morphogenic protein and collagen genes. Laminin and collagen are important components of the blood-brain barrier, and their expression is increased after a stroke [26]. The laminin matrix is important for neuronal survival [27]. The downregulation of laminins was reported to inhibit glioma invasion, metastasis, and angiogenesis [28, 29]. Bone morphogenic proteins are crucial for the development of both the central and peripheral nervous systems in vertebrates, regulating neural stem cell fate and maturation [30, 31]. Their roles and regulation in chemo brain and tumor brain need to be further elucidated, especially given the fact that many of the BMP genes are epigenetically regulated via DNA methylation [32]. Our initial analysis suggested that non-CNS tumor growth led to demethylation of BMP4 promoter in the PFC tissues of tumor-bearing animals as compared to intact ones (Fig. S1). Role of DNA methylation in BMP4 regulation in tumor brain and chemo brain needs to be further substantiated in the large-scale studies. Here, we noted the downregulation of the neurotrophic factors pathway, which was previously reported to be downregulated by chemotherapy agents and radiation [14, 33], and may therefore constitute biomarkers of brain toxicity. In the future, to gain a full understanding of pathways and processes affected in tumor brain and chemo brain, it would be important to conduct a detailed analysis of the brain signalome and entire interactome, using novel platforms such as OncoFinder and iPANDA [34, 35] that allow an in-depth analysis of entire signalome in context of different diseases and conditions [36–40].

An analysis of the mechanisms of tumor brain and chemo brain showed that the growth of TNBC tumors caused oxidative stress in the PFC tissues of PDX mice, which was evidenced by an increase in 8-oxodG levels. Interestingly, PR+BC tumor growth did not cause any significant increase in 8-oxodG levels in the PFC of TumorGraft mice. The 8-oxodG molecule is formed by the action of reactive oxygen species and is a key marker of oxidative DNA damage [20, 41]. We have previously shown that animal exposure to the chemotherapy agents mitomycin C and cyclophospa-mide caused an accumulation of 8-oxo-dG in PFC tissues [14].

Increased levels of oxidative stress and accumulation of 8-oxodG have been reported in brain tumors[42] during neurodegeneration [43], ischemia [44], Alzheimer's disease, amyotrophic lateral sclerosis, Huntington's and Parkinson's diseases, autism, and other pathologies [21, 45, 46]. Elevated levels of 8-oxo-dG caused neuro-degeneration [47], and oxidative stress and oxidative DNA damage have been reported to be hallmarks of brain aging [48, 49].

The presence of 8-oxodG is highly toxic, and cells have an efficient repair mechanism to eliminate oxidative stress by-products via the action of the base excision repair protein OGG1. Alterations in cellular OGG1 levels constitute yet another marker of oxidative DNA damage [21, 50, 51]. Here, we found a significant reduction in the levels of OGG1 in the PFC tissues of PR+BC mice and a trend towards a reduction of OGG1 in the PFC tissues of TNBC-harboring mice. OGG1 is a glycosylase involved in the initial steps of recognition and removal of 8-oxodG [52, 53], and the success of removal of this highly mutagenic and cytotoxic DNA lesion heavily depends on proper OGG1 function. *OGG1* is important for brain development and function, maintenance of neuronal connectivity, and protection against oxidative DNA damage and apoptosis [54–56]. Loss or decrease in OGG1 levels and increase of 8-oxodG in the genome have been reported in cancer, neurodegenerative diseases, autism, and metabolic diseases [21, 53, 57–62], as well as in brain aging [54, 63, 64].

Oxidative stress was previously reported to be associated with aberrant DNA methylation patterns [21]. Another key finding of our study is the decrease in 5-mC, and parallel increases in 5-hmC and the 5-hmC/5-mC ratio in the PFC tissues of tumor-bearing animals as compared to controls. For the first time, we showed that the growth of TNBC and PR+BC tumors caused a profound and significant reduction of 5-mC and an increase in the levels of 5-hmC levels in the PFC tissues of PDX mice. The ratio of 5-hmC/5-mC also increased.

DNA methylation is important for the maintenance of genome stability and gene expression [65, 66]. It regulates a wide array of cellular processes and is vital for brain development and functioning [66]. Altered DNA methylation has been reported in numerous neurological diseases and conditions [67], and global DNA hypomethylation in the brain has been reported to occur upon radiation exposure [68] and chemotherapy treatments.[14] DNA hydroxymethylation is a recently discovered epigenetic modification [66, 69], and 5-hmC is crucial for brain development. It is significantly increased in neurons, whereby hydroxymethylation accounts for up to 40% of all modified CG dinucleotides in the prefrontal cortex [66, 70]. In conjunction with DNA methylation, hydroxyl-methylation regulates tissue-specific gene expression patterns [71]. Altered hydroxymethylation levels have been reported to occur in autism [72], Alzheimer's disease [72], intracerebral hemorrhage [73], and other conditions. Hydroxymethylation levels were affected by proton exposure [68] and chemotherapy [21]. Recent studies showed increases in hydroxymethylation during aging, suggesting that hydroxymethylation might play a role in age-related neurodegeneration [74, 75].

DNA methylation is established and regulated by DNA methyltransferases [69]. We have shown that the growth of malignant non-CNS tumors caused changes in the levels of DNA methyltransferases. These changes can be viewed as protective or compensatory, aimed to restore 5mC losses. Alternatively, decreased levels of DNMTs may be causatively associated with the lessened levels of 5mC. Aberrant levels of DNMTs have been reported to occur in many neurological and psychiatric conditions. They have also been shown to occur upon exposure to radiation and toxic chemicals [14, 76]. The mechanisms of their aberrant expression and the importance of DNMTs in tumor brain and chemo brain should be analyzed in the future.

Similarly, the mechanisms of 5mC loss and 5hmC gain and their functional consequences should be analyzed. Moreover, we studied the global levels of DNA methylation and hydroxymethylation that reflected the net gain or loss across the genome in PFC tissue. The precise locus specificity of the observed changes must be investigated in the future. An analysis of the mechanisms of DNA methylation loss may shed light on potential ways to prevent or mitigate tumor brain and chemo brain. Several studies have shown that alterations in DNA mechanisms led to a reversal of drug resistance in cell line models of breast cancer [77].

The observed loss of DNA methylation may be linked to altered gene expression and genome stability. As such, it would be prudent to link gene expression changes with locus-specific alterations in DNA methylation, as this would allow the establishment of mechanistic links between the two phenomena in the context of tumor brain and chemo brain. Epigenetic marks are tissue-specific, but for diagnostic purposes, the analysis of patient brain tissue is, not possible. Several studies have investigated epigenetic patterns in the brain, blood, and saliva. They reported a high correlation between blood and brain DNA methylation patterns [78–80]. Furthermore, DNA methylation patterns in saliva correlated strongly with DNA methylation patterns in the brain [81]. Therefore, it would be critical to analyze molecular epigenetic changes in the blood of PDX animals and correlate those with brain changes to establish possible mechanisms and the relationship between the two. Blood-based liquid biopsy markers may, therefore, help establish a timeline of changes in tumor brain and chemo brain, as well as for clinically significant biomarkers.

## OUTLOOK

Both chemo brain and tumor brain were first reported in breast cancer; hence, we focused on breast-cancer PDX models. In this study, we analyzed mice that carried PDXs of T4 TNBC and T4 PR+BC tumors. In the future, it would be important to analyze tumor brain as a function of breast tumor type, stage, and grade. Clinical evidence shows that chemo brain occurs in other malignancies, including hematological malignancies, sarcoma, colon, and other cancers [2, 82]. Tt would be prudent to analyze tumor brain in the PDX models of these other cancers. Some changes may be tumor-specific, but some may be common for all tumor brain manifestations.

Changes seen in this study were observed after three weeks of treatment and 3-4 months of tumor propagation. Because of this, it is not possible to pinpoint when the changes occurred, which changes were primary, and which were secondary. It would be important to analyze changes as a function of time. Additionally, we focused on the analysis of molecular changes in the PFC tissues of TumorGraft animals. The PFC has been associated with the execution of functions such as planning, decision-making, behavioural inhibition, and working memory, to name a few [83]. In our earlier study we noted that cytotoxic chemotherapy profoundly affected the PFC [14]. Divided into the medial PFC and orbital prefrontal cortex in rodents, the PFC receives dopaminergic inputs from the ventral tegmental area and connects with virtually all regions of the forebrain. Stress and psychoactive drugs both profoundly alter neuronal morphology in the subregions of the PFC [84]. In a follow-up study we will examine neuronal morphology in the PFCs of intact, tumor-bearing, and chemotherapy-treated TumorGraft mice.

The hippocampus is one of the main sites of adult neurogenesis. In adult mammals, neurogenesis occurs primarily in two germinal zones: the subgranular zone (SGZ) of the DG and the subventricular zone (SVZ) [85]. Chemotherapy is known to affect neurogenesis. In the future, it would be essential to determine molecular manifestation of tumor brain and chemo brain in the hippocampus, and to analyze cell migration, cell number, and the number of newborn neurons in the dentate gyrus. It would also be crucial to correlate molecular and cellular changes with behavioral repercussions. These may serve as foundations for development of novel strategies for prevention and mitigation of both tumor brain and chemo brain. Moreover, chemotherapy may also exert toxic vascular effects, and those need to be further analyzed in the future [86].

While we noticed that the growth of malignant non-CNS tumors caused profound molecular changes in the PFC tissues of TumorGraft mice, chemotherapy-induced changes were rather modest, and no synergistic or additive affects were noted. This is an intriguing and unexpected finding, which, to our mind, may be due to the effectiveness of chemotherapy in reducing tumor growth. Tumor growth caused significant molecular changes in PFC tissue. All of the used chemotherapy regimens caused significant reduction in tumor volume. If tumor growth is an important culprit in tumor and chemo brain, lack of an additive effect of chemotherapy may be explained, at least in part, by tumor volume reduction. In the future, it would be important to analyze tumor brain and chemo brain as a function of chemotherapy effectiveness.

**Figure 7:**
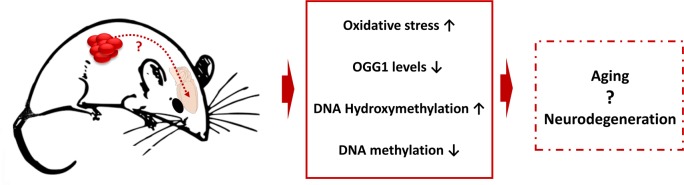
Tumor brain-induced changes may be connected to the aging and neurodegeneration - model scheme

Additionally, in our earlier study we reported that cytotoxic chemotherapy agents mitomycin C and cyclophosphamide affected DNA methylation and caused oxidative stress, and that chemotherapy-induced effects were similar to aging-related processes. Moreover, recent clinical analysis suggested a link between brain aging and cancer treatments [87]. The molecular changes observed in this tumor brain study – altered gene expression, oxidative damage, reduced OGG1 levels, and altered levels of DNA methylation and hydroxymethylation – are known signs of neurodegeneration and brain aging (Fig. 7) [64, 88–90].

The present study suggests links between tumor brain and brain aging, and provides an important roadmap for future analysis. Also, this study lays a foundation for the large-scale analysis of the molecular and cellular mechanisms of tumor brain.

## MATERIALS AND METHODS

### Animal model

Brain tissues of TumorGraft mice were provided by Champions Oncology, Inc. (Baltimore, MD). Patients from the United States diagnosed with triple negative breast cancer (TNBC) and progesterone positive breast cancer (PR+BC) had their tumors engrafted to generate a personalized TumorGraft patient-derived xenograft (PDX) mouse model. In this model, a fresh specimen of the patient's tumor is removed during surgery and fragments of the tumor measuring approximately 4 mm^3^, containing both malignant cells and supportive stromal components, are implanted subcutaneously into the flanks of 6-week-old immunodeficient female mice and propagated as previously described (female-*nu*/*nu* athymic mice; Harlan Laboratories, Indianapolis, IND) [16, 17, 91–93]. Patients provided informed consent documents that followed all federal regulatory requirements and covered the use of tumor material for research purposes. Animal treatments were conducted in accordance with the Institutional Animal Care and Use Committee protocols. Upon propagation when TumorGrafts reached a volume greater than 200 mm^3^, the animals were divided into groups of 3 to 4, and dosing of drugs or drug combinations was implemented according to the individual physician's choice and in consultation with the specific patient. Starting volumes varied between different TumorGraft models because of the individual doubling time. TNBC PDX-bearing TumorGraft animals were treated with Doxorubicin/Paclitaxel/Cyclophosphamide (n=4 treated and 4 untreated), and PR+BC animals with Topotecan (PR+BC/TOP) or Crizotinib (PR+BC/CRIZ) (n=3 treated and untreated). Intact animals (n=3) served as control. Champions Oncology conducted chemotherapy treatments, and all chemotherapy agents were formulated according to manufacturer's specifications. To monitor chemotherapy effects, tumor dimensions were measured twice weekly and tumor volume was calculated as described [16]. In both cases, chemo-therapy applications resulted in successful reduction of tumor growth (data not shown). Animals were euthanized, and the brains were removed from the skulls and immediately flash-frozen in liquid nitrogen and stored in -80C for molecular analysis.

### Gene expression analysis

The prefrontal cortex (PFC) tissues of three–four animals per group were used for the analysis of gene expression profiles. RNA was extracted from PFC tissue using TRIzol® Reagent (Invitrogen, Carlsbad, CA), further purified using an RNAesy kit (Qiagen), and quantified using Nanodrop2000c (Thermo-Scientific). Afterwards, RNA integrity and concentration were established using 2100 BioAnalyzer (Agilent). Sequencing libraries were prepared using Illumina's TruSeq RNA library preparation kits, and global gene expression profiles were determined using the Next 500 Illumina deep-sequencing platform at the University of Lethbridge Facility. Statistical comparisons between the control and exposed groups within each tissue type were performed using the DESeq Bioconductor package (version 1.8.3) and the baySeq Bioconductor package (version 1.10.0). Clustering of the samples was assessed with multi-dimensional scaling (MDS) plots built using the plotMDS function from the edgeR Bioconductor package. MA plots showing the relationship between the average level of expression and the log2 fold change were created for each of the comparisons. The MA-plot is a plot of the distribution of the red/green intensity ratio (“M”) plotted by the average intensity (“A”). Features with a false discovery rate (FDR) < 0.1 (10% false positive rate) were considered differentially expressed between conditions.

The functional annotations of differentially expressed genes were performed using David, GO (Gene Ontology) Elite, and GO-TermFinder [94]. Pathways were visualized using Pathview/KEGG and DAVID bioinformatics platforms DAVID Bioinformatics Resources 6.7 KEGG Pathway platforms [95–97].

### Analysis of 8-oxo-7-hydrodeoxyguanosine, 5-methylcytosine, and 5-hydroxymethylcytosine in DNA

DNA was extracted from PFC tissues using the Qiagen DNeasy Kit. The levels of 8-oxodG, 5mC, and 5hmC in the DNA of mouse PFC tissues were measured by liquid chromatography combined with electrospray tandem mass spectrometry (LC-MS/MS) as previously described [14, 21, 98].

### Analysis of BMP4 promoter methylation

BMP4 promoter methylation was analyzed using the EpiTect Methyl II DNA Restriction Kit and the EpiTect Methyl II PCR Primer Assay for Mouse Bmp4 (CpG Island 103407) (SABiosciences/Qiagen, Toronto, Ontario) following manufacturer's instructions.

### Western immunoblotting

Western immunoblotting was conducted as previously described [14, 33, 76]. In brief, around 50 mg of PFC tissues were sonicated in ice-cold 1% SDS and immediately boiled. Protein concentrations were ascertained using the Bradford assay (BioRad, Hercules, CA). Equal amounts of protein (10-30 μg) were separated by SDS-PAGE into slab gels of 10-15% polyacrylamide and transferred to polyvinylidene difluoride membranes (Amersham Biosciences, Baie d'Urfé, Quebec). The membranes were incubated with primary antibodies against APE1, OGG1, DNMT1, DNMT3A, MeCP2, BMP4, DNMT3B, Laminin 1-2 (1:1000, Abcam), and actin (1:2000, Abcam) overnight at 4°C. Primary antibody binding was detected using horseradish peroxidase-conjugated secondary antibodies and the Enhanced Chemiluminescence Plus System (Amersham Biosciences, Baie d'Urfé, Quebec). Chemiluminescence was detected using a FluorChem HD2 camera with FluorChem software (Cell Biosciences). The membranes were stained with Coomassie blue (BioRad, Hercules, CA) to confirm equal protein loading. Signals were quantified using NIH Image J64 software and normalised relative to actin or Coomassie staining.

### Statistical analyses

Statistical analysis (Student's t-test) was performed using the Microsoft Excel software package.

## SUPPLEMENTARY MATERIAL FIGURE


