# Supplementary material for: Chemo brain or tumor brain - that is the question: the presence of extracranial tumors profoundly affects molecular processes in the prefrontal cortex of TumorGraft mice
Source: Aging (Albany NY). 2017 Jul 29;9(7):1660–75. doi: 10.18632/aging.101243 (PMC5559168; doi:10.18632/aging.101243)
Supplement: Supplementary file 1 [file aging-09-1660-s001.pdf]

SUPPLEMENTARY MATERIAL

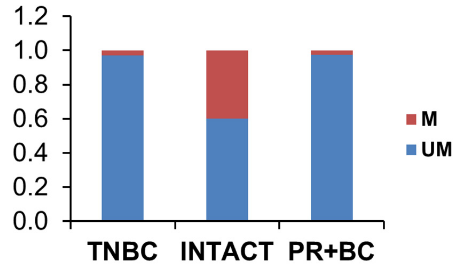

Supplementary Figure 1. Levels of BMP 4 promoter methylation. UM-unmethylated, M-methylated.
